# Media device ownership and media use: Associations with sedentary time, physical activity and fitness in English youth

**DOI:** 10.1016/j.pmedr.2016.05.013

**Published:** 2016-06-03

**Authors:** Gavin R.H. Sandercock, Mohammed Alibrahim, Mark Bellamy

**Affiliations:** a University of Essex, Colchester, UK; b Performance Technology Ltd. Hereford, UK

**Keywords:** Television, Cell phones, Text messaging, Social media

## Abstract

The aim of this study was to determine whether ownership and use of electronic media were associated with sedentary time and cardiorespiratory fitness (fitness) in youth. We also aimed to determine if associations were independent of physical activity (PA).

Fitness was measured using the 20 m shuttle-run. PA, sedentary time, ownership of media devices and media use were self-reported.

Participants (n = 678, age 10–15 years) reported daily sedentary time of 620 (± 210) min. Forty-one percent of participants had low PA and 50.4% had low fitness.

Higher weekend sedentary time was associated with low fitness in girls (*p* = 0.005) and boys (*p* < 0.001) and remained significant when adjusted for PA in the latter (*p* = 0.006). Using social media was associated with higher sedentary time in both sexes and low fitness in girls. High sedentary time was more likely (OR = 5.3, 95%CI: 2.0–14.4) in boys who owned game consoles. Low fitness was more likely in boys who owned digital/satellite TV receivers (OR = 1.8, 95%CI: 1.8–3.2).

Schoolchildren spent > 10 h or ~ 85% of each waking day sedentary. Use of social media was associated with higher sedentary time in both sexes and with low fitness in girls. Reducing social media use in youth offers one potential target for intervention. Behaviours associated with sedentary time differed from predictors of low fitness. The complex and often sex-specific interactions identified between sedentary time, PA and fitness suggest the need for carefully targeted interventions to reduce sedentary time and improve fitness in English youth.

## Highlights

• English schoolchildren spend most of their waking time engaged in sedentary behaviours.
• Physical activity and sedentary time are independently associated with low fitness in boys.
• Owning a game console is associated with higher sedentary time in boys.
• Use of social media is associated with higher sedentary time in boys and girls.
• Owning a digital/satellite TV receiver doubles the risk of low cardiorespiratory fitness in boys.

## 1 Introduction

The proliferation of new media and increased availability via multiple devices, quickly render obsolete research examining the associations between media and health. Early studies of children's sedentary behaviours concerned TV viewing (Biddle et al., 2009a, Eisenmann et al., 2005) while later studies concerned ‘screen-time’(Ekelund et al., 2006, Ogunleye et al., 2012) but neither measures accurate estimates overall sedentary behaviour (Biddle et al., 2009a). These studies were of great value as they identified that children engaged in excessive screen-time were often inactive, unfit and in poor metabolic health (Ekelund et al., 2006). It remains unclear whether screen time is negatively associated with health simply because it displaces physical activity (PA) or whether there is a more complex relationship (Cummings and Vandewater, 2007).

Physical activity (PA) promotes health through preferential adaptation of multiple physiological systems (Warburton et al., 2006). One such adaptation is quantifiable through the assessment of cardiorespiratory fitness (herein ‘fitness’) (Williams, 2001). Objective measures of fitness are more powerful predictors of health than measures of PA (Williams, 2001) and may even provide better estimate of habitual PA than self-report (Swift et al., 2013).

Sedentariness and PA are discrete constructs and there is some evidence from objective measures of both that they are independently associated with children's fitness (Santos et al., 2014) (Martinez-Gomez et al., 2011). Data from the HELENA study (Martinez-Gomez et al., 2011) found a negative association between sedentariness and fitness of inactive girls. However, no significant associations were seen in girls achieving 60 min daily PA (active) no boys. Objective measures accurately quantify the time children spend being sedentary but lack the contextual information regarding the behaviours comprising sedentary time available through self-report.

One such measure, screen-time, has also been identified as a predictor of children's fitness independent of PA (Arango et al., 2014, Sandercock and Ogunleye, 2013). Assessing screen-time does not capture the entirety of sedentary behaviours (Olds et al., 2010, Pate et al., 2011) in the way that time-use diaries can (Biddle et al., 2009a). It is ten years since the completion of the landmark study detailing British children's sedentary (Biddle et al., 2009a) in which time there have been many innovations in media and changes in media consumption habits (Ofcom, 2015).

The aims of this study were threefold. First, we sought to identify the media devices that are most-commonly owned, the prevalent patterns of media-use in youth and how they are related to sedentary time. Second, we aimed to determine whether sedentary time and PA were independently associated with fitness. Finally, we aimed to identify associations between media device ownership, media-use and fitness.

## 2 Methods

Ethical approval was granted by the University Ethics Committee. Written, informed school, parental and pupil consent were also obtained before testing. Data were collected from May–June 2014 in pupils attending six schools that had previously participated in the East of England Healthy Hearts Study (Voss and Sandercock, 2010). Schools were recruited to produce a regionally representative sample at school level based on geographical (rural, urban), school type, (junior or high school) and area-level deprivation. As the area is relatively affluent we purposefully recruited schools drawing students entirely (junior school), or partly (high schools) from deprived areas.

The final sample comprised four junior schools (one rural, one deprived), one urban high school and one high school with a mixed (rural and urban) catchment. We measured one class from grade 6 in three junior schools and two classes from the school in a more-deprived area. We also sampled two classes from each Grade (7–11) of both high schools providing a potential sample of approximately *n* = 750. Parental consent could not be obtained for *n* = 46 (6%) pupils, the remainder of missing cases were due to absenteeism or non-attendance in PE class. We measured *n* = 704 pupils but data were incomplete in a further *n* = 26 (3%) of participants due to injury, incomplete or illegible questionnaires.

### 2.1 Demographic information

Date of birth was used to calculate decimal age. Home postcodes provided measures of area-level deprivation via the English Indices of Deprivations (EID) (Department for Communities and Local Government, 2007).

### 2.2 Media device ownership and media use

Full details of the development of this reporting tool are given in the supplementary materials along with the questionnaire. Participants indicated media devices they owned ‘*media ownership*’ and media use behaviours ‘*media use*’ from a menu of options. The numbers of daily email, text and instant messages sent were recorded on a categorical scale with responses from ‘zero’ through > 50. Participants also reported the sum of these and any other messages sent each day (*Total Messaging*).

### 2.3 Physical activity

Participants completed the PAQ-A (Janz et al., 2008, Kowalski et al., 1997) a 7-day PA recall tool with acceptable validity for this age-group (Janz et al., 2008, Kowalski et al., 1997), normative values and criterion-referenced cut-offs for low PA (Voss et al., 2013).

### 2.4 Cardiorespiratory fitness

Cardiorespiratory fitness ‘*fitness*’ was assessed using the 20 m shuttle-run test. Performance was recorded as running speed at the final completed level. Running speed was converted to an estimate of VO_2peak_ (ml kg^− 1^ min^− 1^) using a standard equation (Leger et al., 1988) and participants grouped using age- and sex-specific cut-offs (Bell et al., 1986). Participants below the cut-off values were categorised as having low cardiorespiratory fitness; ‘*Low Fitness*’.

### 2.5 Sedentary time

To limit participant burden, we used a menu-based approach in which items were restricted to behaviours that contribute significantly to sedentary time (Biddle et al., 2009b) (Olds et al., 2010). We were careful to ensure there was no direct overlap between the items contributing to sedentary time and media-use behaviours listed. Total weekday and weekend sitting time was calculated by summing time spent on each activity. Average 7-day sitting time was calculated as: (Weekday Sedentary Time × 0.71) + (Weekend Sedentary Time × 0.29). As there are no quantitative recommendations relating to sedentary time in England, cut-offs for high and low levels of sedentary time were therefore created based on sex-specific median splits.

### 2.6 Data analysis

We compared total reported sedentary time, PA and fitness (Table 1) between boys and girls using (independent *t*-tests). Continuous variables that were dichotomized (low fitness, low PA) and those already categorical in nature (media ownership) were analysed using χ^2^. As the media use questions that had quantitative, but categorical response options data were treated as ordinal and were described using the median and inter-quartile range (IQR). Between-sex differences were analysed using Mann–Whitney *U-*tests.

Intraclass correlation coefficients (ICC) showed sedentary time, PA and fitness to be clustered within schools (ICC = 0.18–0.24). To control for school-level clustering mixed, multilevel regression analyses were used with ‘school’ as a random factor.

Dichotomisation of continuous data (particularly by median split) may lead to residual confounding and reduced statistical power (Altman and Royston, 2006). When investigating predictors of high sedentary time (Table 3) mixed linear regression was used prior to logistic analyses. Stepwise linear analysis was used to identify independent predictors of sedentary time. Independent, non-collinear (Variance Inflation Factor < 2.5) predictors were then used in subsequent logistic analyses. Separate analyses were performed for girls and boys.

To identify elements of *media ownership* and *media use* associated with high sedentary time potential predictors were entered into a mixed logistic regression model adjusted for passive school transport (Aggio et al., 2012).

Mixed logistic regression was also used to determine if sedentary time and PA were independently associated with low fitness. Expressing sedentary time and PA as continuous variables, we calculated univariate associations with low fitness (Table 4, upper panel). We then created multivariate models including both weekday and weekend sedentary time (Model^1^). These were next adjusted for age and deprivation (Model^2^) and then PA (Model^3^).

Following preliminary linear analysis, mixed logistic regression was used to identify if specific elements of media ownership and media use were independently associated with low fitness. Predictors were initially adjusted for one another (Model^1^). Next, estimates were adjusted for age and deprivation (Model^2^) then additionally for PA (Model^3^). All analyses were performed using STATA Version 14.0 (StataCorp Ltd., TX. USA) (Table 5).

## 3 Results

There were no significant differences in the sedentary time reported by boys and girls but boys were more active and fitter than girls (Table 1). When age- and sex-specific cut-offs were applied there was no significant difference in the prevalence of low fitness between boys (41%) and girls (43%).

### 3.1 Sedentary time

Detailed composition of weekday sedentary time is given in supplementary materials (Fig. 1). School was the predominant component of sedentary time in both sexes. The contribution of passive transportation, talking and eating (~ 15%) and watching television (~ 15%) was comparable between sexes but girls spent 40% more time completing homework than boys. Using a computer accounted for 24% of sedentary time in boys, was 30% higher than in girls.

### 3.2 Media ownership and media use

Media ownership and media use behaviours are shown in Table 2. Additional television ownership (and a device to receive, record and play content) was the most-commonly reported media ownership across both sexes. More boys (90%) owned a standard game console than girls (54%). Fewer participants of either sex (boys, 52%; girls, 48%) owned active game consoles. More boys than girls owned desktop computers but there were no significant between-sex differences in ownership of laptop or tablet computers.

Two thirds of schoolchildren owned a smart phone and an additional 40% owned a standard mobile phone. While 20% of girls and 17% of boys owned more than one phone, only 10% of boys and 5% did not own their own mobile phone.

Over three-quarters of participants used social media; this media use was marginally more common in girls than boys. Total messaging was higher (p < 0.001) in girls (median 50, [10–80]) than boys (20, [5–35]). Total Messaging exceeded the sum of email, Text and Instant Messages reported.

### 3.3 Media ownership and media use: associations with sedentary time

Table 3 shows that the results of regression analyses describing associations between media ownership and media use were associated with sedentary time (linear) and high sedentary time (logistic). Linear and logistic models broadly agreed with one another. Linear analysis showed owning a game console was associated with 170 min higher sedentary time. Logistic analysis confirmed boys who owned game consoles were more likely (OR = 5.3, 95%CI: 2.0–14.4) to have high sedentary time. Owning a desktop computer was associated with 51 min additional sedentary time (linear model) and a (60%) greater likelihood of high sedentary time (logistic model). Linear analysis showed ‘time spent using social media’ rather than the ‘use of social media’ (binary) to be more strongly associated with sedentary time, so only the former, continuous variable was used in logistic analysis.

In girls, each minute spent using social media was associated with an additional 1.5 min daily sedentary time (*β* = 1.51) and a 2% increase in the likelihood (OR = 1.02, 95%CI: 1.01–1.03) of high sedentary time (logistic analysis). Linear analysis suggested that owning an Active Game Console was associated with 85 min, (*p* < 0.001) additional sedentary time and an increased likelihood of high sedentary time (OR = 1.72, 95%CI: 0.91–3.25, *p* = 0.096). Girls who owned a Blu-Ray/DVD Player were less likely to have high sedentary time.

### 3.4 Sedentary time, physical activity and low fitness

Mixed linear regression showed that higher weekday sedentary was associated with an increased likelihood of low fitness in girls (*p* = 0.009) and boys (*p* = 0.006). In a separate analysis, weekend sedentary time was negatively associated with PA (*p* < 0.001 in both sexes). When combined into a single model, weekend sedentary time was a significant predictor of low fitness (Model^1^); this association was independent of age, deprivation (Model^2^) and PA (Model^3^). Weekend sedentary time was a significant predictor in girls (Model^1^) and was also independent of age and deprivation (Model^2^). After adjusting for PA (Model^3^) the association between weekend sedentary time and low fitness became non-significant (*p* = 0.489) but the strength of association with weekday sedentary time increased, approaching statistical significance (*p* = 0.095).

### 3.5 Associations between media device ownership, media-use and fitness

Table 5 shows boys who owned a digital/satellite TV-receiver were twice as likely to have low fitness (OR = 2.12, 95%CI: 1.31–3.94). This association was attenuated slightly (OR = 1.84, 95%CI: 1.04–3.24) but remained statistically significant (*p* = 0.035) after adjusting for age, deprivation and PA.

Time spent on social media was associated with a greater likelihood of girls having low fitness (OR = 1.006, 95%CI: 1.001–1.011). After adjusting for age, deprivation and PA, time spent on social media was no longer significantly associated with low fitness. Independent of age and deprivation, girls who owned a desktop computer were less likely to be unfit (OR = 0.37, 95%CI: 0.16–0.89) but Model^3^ suggested that this association was not independent of PA (OR = 0.54, 95%CI: 0.21–1.40).

## 4 Discussion

These data provide a contemporary snapshot of media ownership and media use in English youth. Our aims were to identify the contribution of electronic media to sedentary time and investigate whether media ownership or use were associated with cardiorespiratory fitness.

### 4.1 Sedentary time, physical activity and fitness

The present sample reported very high levels of sedentary time during weekdays. Assuming schoolchildren (mean age 13 years) sleep for 9 h, provides 15 waking hours each day. Mean weekday sedentary time was comparable in boys (767 min) and girls (739 min). The overall sample mean (755 min) suggests that schoolchildren spent 12.5 h or 83% of weekday waking time being sedentary. This was confirmed by 7-day accelerometry in a subgroup (*n* = 76) of participants (Supplementary materials Table S1). Supplementary Fig.1a–1b shows the important contribution of school to weekday sedentary time. School has received surprisingly little attention from research (Biddle et al., 2014, Sallis et al., 2000, van Sluijs et al., 2010) much of which has tended to focus on screen-based media but may already be out-dated due to rapid advancement of technology and changing patterns of media consumption.

### 4.2 Media device ownership and use

One third of participants owned smart phones but there is a paucity of data regarding these devices and whether they are associated with PA. These data suggest that adolescents spend a significant amount of time sending and receiving messages. National data show that 63% of UK teenagers send messages daily (Ofcom, 2015). We found that most girls sent 50 messages per day, although this may be over-reported due to the categorical nature of the responses we supplied. A negative association between heavy mobile phone use (including high messaging behaviour) and PA has been reported in college-age students (Lepp et al., 2013). There were, however, no studies in children published at time of writing. Future research into this behaviour would need to account for the recent decline in text-based messaging as it is now being replaced by face-to-face communication via the internet (Ofcom, 2015).

### 4.3 Media ownership, media use and sedentary time

Participants owned 7–10 media devices with half owning an active game console. While there may be a potential for active gaming to reduce sedentary time and promote physical (Biddiss and Irwin, 2010), we found that girls owning and active console reported 85 min (95%CI: 39–132) more daily sedentary time and had an increased (OR = 1.71, 95%CI: 0.91–3.25) likelihood of high sedentary time. We cannot infer causation from this finding and owning one of these relatively new devices may act as a surrogate measure for overall device ownership. Unplanned *post hoc* analysis showed that girls owning active consoles also owned (median 5, IQR 4–7) more than twice as many media devices as girls who did not (median 2, IQR 1–4).

Historically, television viewing has been the predominant media use reported in investigations of children's sedentary time (Biddle et al., 2009a). Studies have shown that ownership of a bedroom television is associated with higher sedentary time (van Sluijs et al., 2010) and lower physical activity levels (Gorely et al., 2004, Salmon et al., 2011) although results have been mixed (Delmas et al., 2007, Salmon et al., 2011). The numbers of boys (83.4%) and girls (68.6%) who owned a TV are above those reported in other European children (van Sluijs et al., 2010). Since 2010 media consumption habits have changed dramatically with 33% of teenagers now watching (74 min/day) TV via their phone (Ofcom, 2015). The lack of any significant associations between TV ownership and sedentary time may indicate a decline in the relative contribution of television to sedentary behaviour. Boys were more likely to own a game console the device most strongly associated with high sedentary time. Game consoles represent a potential target for reducing sedentary time in boys.

Time spent using social media was the only predictor of higher sedentary time common to both sexes and the strongest correlate of high sedentary time in girls (Table 3). Messaging behaviour was collinear with time spent using social media with girls reporting more time spent engaged in both behaviours. Sending fifty messages per day (*n* = 3 per waking hour) appears typical for girls of this age (Lenhart et al., 2010) and there is likely to be some overlap between these behaviours due to ‘messaging’ capacity within social media. The integration of the ‘*messenger*’ app into Facebook during our study exemplifies some of the difficulties when studying rapidly changing media.

### 4.4 Associations between sedentary time, physical activity and fitness

Associations between high screen-time and low fitness have been reported previously (Marques et al., 2015, Sandercock and Ogunleye, 2013). Studies assessing the association between fitness and more-complete measures of sedentary behaviour have produced mixed findings regarding their independence from PA (Marques et al., 2015, Martinez-Gomez et al., 2011, Santos et al., 2014). The association between weekend sedentary time and fitness was stronger than that for weekday sedentary time. The association was stronger and independent of PA in boys. Both findings are likely due to the greater variance in weekend compared with weekday sedentary time and in boys’ sedentary time compared with girls’. Boys reported more weekend sedentary time than girls despite being more physically active (Ekelund et al., 2006, Martinez-Gomez et al., 2011, Sandercock et al., 2012). This might suggest that boys would benefit more than girls from interventions designed to decrease weekend sedentary time. As they report less sedentary time but also tend to be less active (Sandercock and Ogunleye, 2013, Santos et al., 2014), girls may benefit more from interventions promoting increased PA (Martinez-Gomez et al., 2011).

Interventions must be targeted if they are to be effective in changing complex behaviours. In boys owning a desktop computer was associated with higher sedentary time but not with low fitness; whereas boys owning a digital/satellite TV receiver were more likely to be unfit. These devices give children control over programming and may be the contemporary equivalent of a ‘bedroom TV’ (Delmas et al., 2007).

Time spent using social media was the only behaviour associated with sedentary time in both sexes and with low fitness in girls. Access to social media continues to expand through multiple platforms and devices (Ofcom, 2015). The potentially negative effects of social media on children's mental health have been investigated but research concerning the physical health remains scarce (Richards et al., 2015).

### 4.5 Limitations

The observational nature of this study presents several limitations but the inability of this non-experimental design to discern the direction of causality between media use and sedentary behaviours is primary. The major limitation of this study is the use of self-reported sedentary time and PA. The former was necessary to gain contextual information regarding sedentary behaviours. We also made objective measures of sedentary behaviour in a subsample of *n* = 78 children, these suggested that weekday estimates of sedentary time were credible but that there was some under-reporting of weekend sedentary time (Supplementary materials Table S1). For the latter we used an anglicized version (Voss et al., 2013) of a validated questionnaire (Janz et al., 2008, Kowalski et al., 1997). We also used objective measures of fitness as an outcome variable as fitness is more-strongly and independently associated with health than PA (Williams, 2001). As data were collected in the summer months they provide no insight into seasonal variations in physical activity or sedentary behaviours. We would likely observe lower PA and possibly higher sedentary time data were collected in winter when there are fewer daylight hours, lower temperatures and higher rainfall.

Finally, our focus group volunteers were older than our target population. While the focus groups greatly increased the ‘credibility’ of items, future studies would benefit from recruiting individuals of the same age as the study population.

## 5 Conclusions

English youth have high levels of sedentary time, low levels of PA and there is a high prevalence of low cardiovascular fitness. School and use of electronic media account for much of sedentary time, which is independently associated with low fitness, particularly in boys. The composition of sedentary time differs greatly between boys and girls evidencing the necessity of targeted approaches to reducing this potentially un-healthful behaviour. Our data suggest that such targeted approaches might include attempts to reduce the time both sexes spend using social media or discouraging boys' ownership of TV receiving/recording devices.

## Transparency document

Transparency document Image 1

## Conflict of interest

The authors declare that there is no conflict of interest.

## Funding

This research was funded by *PROVIDE* Public Health, Essex. Grant DB0500H1.

## Figures and Tables

**Table 1:** Sedentary time, physical activity and cardiorespiratory fitness in English schoolchildren (age 10–16 years).

	Boys (*n* = 370)	Girls (*n* = 308)	Mean difference (95%CI)	*t-*Test or *χ*^*2*^, *p*-value
Mean (SD)	Mean (SD)
Age (years)	13.5 (0.8)	13.4 (0.8)	0.1 (− 0.6–0.4)	*t* = 0.78, *p* = 0.695
Area-level deprivation (EID score)	12.9 (4.8)	13.0 (4.5)	0.8 (− 1.4–2.0)	*t* = 0.61, *p* = 0.879
Ethnicity (% white British)	95.2% (*n* = 356)	92.9% (*n* = 286)	–	*χ*^*2*^ = 1.81, *p* = 0.118
Weekday sedentary time (min)	767 (290)	739 (261)	28 (− 14–69)	*t* = 1.30, *p* = 0.191
Weekend sedentary time (min)	258 (212)	243 (170)	14 (− 14–15)	*t* = 0.94, *p* = 0.291
Daily sedentary time (min)	627 (231)	612 (191)	15 (− 18–48)	*t* = 0.89, *p* = 0.373
7-Day physical activity (PAQ-A)	2.92 (0.72)	2.55 (0.58)	0.36 (0.26–0.46)	*t* = 1.72, *p* = 0.017
Low physical activity^a^	47% (*n* = 170)	54% (*n* = 163)	–	*χ*^*2*^ = 1.85, *p* = 0.112
Cardiorespiratory fitness (ml kg^− 1^ min^− 1^)	43.4 (5.1)	38.2 (5.4)	5.2 (4.4–6.0)	*t* = 12.3, *p* < 0.001
Low cardiorespiratory fitness^b^	40.9% (*n* = 126)	42.9% (*n* = 129)	–	*χ*^*2*^ = 1.85, *p* = 0.112

Legend: EID — English Indices of Deprivation; PAQ-A (Physical Activity Questionniare for Older Children: 1–5 score), (Kowalski et al., 1997).

a Based on cut-offs of Voss et al. (2013).

b Based on cut-offs of Bell et al. (1986). All data collected in summer term (May–June) 2014 at *n* = 6 schools, Essex, UK.

**Table 2:** Prevalence of media device ownership and use in male and female English high school pupils.

	Boys	Girls	χ^2^*p*-value
Total	54.5% (*n* = 370)	45.5% (*n* = 308)	*χ*^*2*^ = 6.40, *p* = 0.011
Additional television^a^	83.4% (*n* = 312)	68.6% (*n* = 210)	*χ*^*2*^ = 20.6, *p* < 0.001
Blu-ray/DVD player^a^	57.1% (*n* = 212)	53.0% (*n* = 168)	*χ*^*2*^ = 0.31, *p* = 0.164
Additional television receiver^a^	37.4% (*n* = 140)	28.6% (*n* = 68)	*χ*^*2*^ = 5.80, *p* < 0.010
Game console^a^	90.4% (*n* = 336)	54.0% (*n* = 168)	*χ*^*2*^ = 107, *p* = 0.001
Active console^a^	52.2% (*n* = 194)	48.1% (*n* = 148)	*χ*^*2*^ = 0.99, *p* = 0.360
Desktop computer^a^	30.1% (*n* = *11*2)	16.4% (*n* = 50)	*χ*^*2*^ = 17.1, *p* < 0.001
Laptop computer^a^	53.8% (*n* = 200)	66.7% (*n* = 204)	*χ*^*2*^ = 11.6, *p* < 0.001
Tablet computer^a^	42.8% (*n* = 160)	43.5% (*n* = 134)	*χ*^*2*^ = 0.08, *p* = 0.825
Smart phone^a^	65.8% (*n* = 245)	67.5% (*n* = 208)	*χ*^*2*^ = 0.28, *p* = 0.625
Other mobile phone^a^	41.1% (*n* = 149)	47.7% (*n* = 147)	*χ*^*2*^ = 2.99, *p* = 0.049
No mobile phone	10.3% (*n* = *38*)	5.2% (*n* = 16)	*χ*^*2*^ = 8.91, *p* = 0.003
More than one mobile phone	16.8% (*n* = 62)	20.3% (*n* = 62)	*χ*^*2*^ = 1.37, *p* = 0.272
Use of social media	76.2% (*n* = 282)	77.8% (*n* = 240)	*χ*^*2*^ = 0.23, *p* = 0.648


Continuous measures	Boys		Girls		Mann–Whitney U-test
Median (IQR)	Mode	Median (IQR)	Mode
Total media device access^b^	8 (7–10)	10	8 (7–10)	10	*U* = − 2.4, *p* = 0.017
Total media device ownership^a^	6 (4–7)	6	6 (3–7)	6	*U* = − 3.7, *p* = 0.002
emails (per day)	0 (0–10)	0	0 (0–10)	0	*U* = − 0.5, *p* = 0.550
Instant messages (per day)	5 (0–20)	5	10 (5–50)	40	*U* = − 4.8, *p* < 0.001
Text messages (per day)	10 (5–20)	5	10 (5–20)	10	*U* = − 3.9, *p* < 0.001
Total messages (per day)^c^	20 (5–35)	10	50 (10–80)	50	*U* = − 4.1, *p* < 0.001
Time using social media (min/day)	30 (30–60)	30	60 (30–90)	60	*U* = − 3.8, *p* < 0.001

a Defined as having sole use of a device that is usually kept in participants' bedroom; whether the device was received as a gift or purchased by the participant.

b Defined as having access to a device shared with other family members.

c Total messages per day includes sum of all messaging media listed plus any additional messaging behaviours. All data collected in summer term (May–June) 2014 at *n* = 6 schools, Essex, UK.

**Table 3:** Media device ownership and media use: associations with daily sedentary time (Linear Model^1^) and with high sedentary time (Logistic Model^2^).

	Boys				Girls		
Media device ownership or media use	*β*	(95%CI)	*p*-Value	Media device ownership or media use	*β*	(95%CI)	*p*-Value
Linear Model^1^							
Games console	170.1^a^	(75.1–266)	< 0.001	Social media (min·day^− 1^)	1.51^a^	(0.79–1.68)	< 0.001
Social media (min·day^− 1^)	0.83^a^	(0.22–1.44)	0.008	Active game console	85.4^a^	(38.6–132)	< 0.001
Desktop computer	50.9^b^	(− 3.65–105.0)	0.067	Smart phone	− 76.6^a^	(− 124 to − 28.7)	0.002
–		–		Blu-Ray/DVD player	− 49.8^a^	(− 90.7 to − 4.94)	0.030
Passive school travel	42.2	(− 11.4 to 95.8)	0.122	Passive school travel	31.3	(− 13.5–75.7)	0.167
Age	10.6	(− 10.9 to 32.2)	0.333	Age (years)	29.5^a^	(12.4–46.6)	0.001
Deprivation (EID)	− 1.70	(− 7.16 to 3.76)	0.540	Deprivation (EID)	− 3.68	(− 12.4 to − 46.6)	0.136

	OR	(95%CI)	*p*-Value	Media device ownership or media use	OR	(95%CI)	*p*-Value

Logistic Model^2^							
Game console	5.32^a^	(1.97–14.4)	< 0.001	Social media (min·day^− 1^)	1.02^a^	(1.01–1.03)	< 0.001
Social media (min·day^− 1^)	1.004	(0.998–1.01)	0.155	Active games console	1.72^b^	(0.91–3.25)	0.096
Desktop computer	1.64^b^	(0.99–2.72)	0.057	Smart phone	0.76	(0.40–1.45)	0.145
Blu-Ray/DVD player	0.48	(0.26–0.90)	0.021
Passive school travel	1.62^a^	(1.00 to 2.65)	0.050	Passive school travel	2.84^a^	(1.49–5.43)	0.002
Age	1.14	(0.93 to 1.41)	0.206	Age (years)	1.18	(0.94–1.50)	0.146
Deprivation (EID)	0.96	(0.92 to 1.01)	0.144	Deprivation (EID)	0.96	(0.90–1.04)	0.365

Legend: EID — English Indices of Deprivation; OR — Odd Ratios, CI — Confidence Intervals; *β* — coefficients are unstandardized. Model^1^ — all variables were statistically significant, non-collinear (Variance Inflation Factor < 2.5) predictors of mean daily (weekday and weekend) sedentary time (min·day^− 1^) were identified using linear regression (Model^1^). Coefficients are for mixed linear regression analysis (Enter) with forced entry of: Passive school transport, Age and Deprivation. Model^2^ — Mixed logistic regression (Enter Method) predicting high sedentary time (sex-specific median splits); Referent category — low sedentary time. Initial variables entered on the basis of statistical significance and non-collinearity in Model^1^; Predictors reported as OR (95%CI). All ORs are mutually adjusted for; one another, Passive school transport, Age and Deprivation. All data collected in summer term (May–June) 2014 at *n* = 6 schools, Essex, UK.

a Statistically significant predictor of high sedentary time.

b Borderline statistical significance but likely important predictor based on OR and 95%Confidence Intervals.

**Table 4:** Sedentary time and physical activity: univariate and multivariate associations with low cardiorespiratory fitness in English schoolchildren.

Univariate	OR (95%CI)	*p*-Value	OR (95%CI)	*p*-Value	OR (95%CI)	*p*-Value
*Boys*						
Weekday (min)	1.001					
(1.0003–1.0020)	*p* = 0.006				
Weekend (min)			1.003			
		(1.002–1.004)	*p* < 0.001		
Physical activity (PAQ score)					0.148	
				(0.095–0.231)	*p* < 0.001
School (clusters)	0.244		0.312		0.192	
(0.038–1.542)		(0.058–1.682)		(0.035–1.044)	

*Girls*						
Weekday (min)	1.001					
(1.0004–1.004)	*p* = 0.009				
Weekend (min)			1.003			
		(1.002–1.005)	*p* < 0.001		
Physical Activity (PAQ 1–5)					0.149	
				(0.142–0.375)	*p* < 0.001
School (clusters)	0.173		0.226		0.174	
(0.037–0.816)		(0.051–1.024)		(0.034–0.883)	


Multivariate	Model^1^		Model^2^		Model^3^	
OR (95%CI)	*p*-Value	OR (95%CI)	*p*-Value	OR (95%CI)	*p*-Value
*Boys*						
Weekday (min)	1.0004		1.0003		1.0003	
(0.999–1.001)	*p* = 0.376	(0.999–1.001)	*p* = 0.406	(0.998***–***1.001)	*p* = 0.953
Weekend (min)	1.003		1.004		1.002	
(1.002–1.004)	*p* < 0.001	(1.002–1.005)	*p* < 0.001	(1.001–1.004)	*p* = 0.006
Age (years)			0.702		0.723	
		(0.574–0.859)	*p* < 0.001	(0.581–0.899)	*p* = 0.004
Deprivation			0.026		1.007	
		(0.979–1.082)	*p* = 0.254	(0.953–1.061)	*p* = 0.793
Physical activity (PAQ 1–5)					0.044	
				(0.113–0.290)	*p* < 0.001
School (clusters)	0.316		0.364		0.596	
(0.060–1.67)		(0.109***–***2.12)		(0.127–2.802)	

*Girls*						
Sedentary time	1.001		1.0005		1.001	
Weekday (min)	(0.999–1.002)	*p* = 0.120	(0.999–1.002)	*p* = 0.429	(0.999*–*1.002)	*p* = 0.095
Sedentary time	1.003		1.0003		1.001	
Weekend (min)	(1.001–1.005)	*p* < 0.001	(1.0002***–***1.005)	*p* = 0.005	(.997–1.002)	*p* = 0.489
Age (years)			1.457		1.405	
		(1.172–1.811)	*p* < 0.001	(1.119–1.76)	*p* = 0.003
Deprivation (EID)			.947		0.962	
		(0.891–1.006)	*p* = 0.079	(0.902–1.026)	*p* = 0.244
Physical activity (PAQ score, 1–5)					0.274	
				(0.158–.0477)	*p* < 0.001
School (clusters)	0.206		0.152		0.134	
(0.044–0.951)		(0.026–1.018)		(0.0173–1.039)	

Legend: Mixed multilevel regression coefficients reported as Odd Ratios (OR) and 95% Confidence Intervals (95%CI). ORs are for likelihood of Low cardiorespiratory fitness. Low fitness defined using cut-offs of Bell et al. (1986). Analysis performed in STATA Version 14.0 with ‘School’ as a random factor. All data collected in summer term (May–June) 2014 at *n* = 6 schools, Essex, UK.

**Table 5:** Media device ownership and media use as predictors of low cardiorespiratory fitness in English schoolchildren (10–15 years).

	Model^1^	Model^2^			Model^3^	
OR (95%CI)	*p-*Value	OR (95%CI)	*p-*Value	OR (95%CI)	*p-*Value
*Boys*						
Owning satellite or digital TV receiver	2.12 (1.31–3.94)	*p* = 0.004	2.33 (1.42–3.83)	*p* = 0.002	1.84 (1.04–3.24)	*p* = 0.035
Age (years)			1.20 (0.99–1.45)	*p* = 0.068	1.20 (0.97–1.49)	*p* = 0.089
Deprivation (EID)			0.96 (0.92–1.01)	*p* = 0.164	0.98 (0.93–1.04)	*p* = 0.065
Physical activity (PAQ score 1–5)					0.15 (0.09–0.24)	*p* < 0.001
Schools (clusters)	5.49 (1.77–17.04)		5.28 (1.71–16.33)		9.64 (4.46–26.8)	

*Girls*						
Social media (min·day^− 1^)	1.006 (1.001–1.011)	*p* = 0.002	1.006 (0.999–1.009)	*p* = 0.059	1.004 (0.998–1.008)	*p* = 0.108
Owning desktop computer	0.38 (0.17–0.90)	*p* = 0.027	0.37 (0.16–0.89)	*p* **=** 0.026	0.54 (0.21–1.40)	*p* = 0.199
Age (years)			1.65 (1.28–2.12)	*p* < 0.001	1.51 (1.16–1.98)	*p* = 0.002
Deprivation (EID)			0.95 (0.88–1.02)	*p* = 0.164	0.96 (0.89–1.04)	*p* = 0.281
Physical activity (PAQ score 1–5)					0.28 (0.16–0.51)	*p* < 0.001
Schools (clusters)	5.60 (2.03–15.4)		5.54 (1.99–15.4)		5.16 (1.84–14.5)	

Legend: EID — English Indices of Deprivation; OR — Odd Ratios and 95% Confidence Intervals (95%CI) — Multilevel mixed Binary logistic regression (Enter) with coefficients reported as adjusted OR (95%CI). OR are for likelihood of low fitness — cardiorespiratory fitness value below cut-offs of Bell et al., (1986). Analysis performed in STATA Version 14.0; with School as random effects factor to adjust for clustering. Referent category — Good Cardiorespiratory Fitness (above cut-offs of Bell et al., 1986) and non-ownership of media devices listed. Social media — Time spent using social media each day, expressed as a continuous variable (min·day^− 1^). Model^2^ adjusted for Age (years) and Deprivation (continuous variables). Model^2^ adjusted for Age, Deprivation and Physical Activity (PAQ-Score, Kowalski et al., 1997). Initial variables selected on the basis of statistically significant linear association with fitness expressed as age- and sex-specific z-score (analysis not shown) and on basis of non-collinearity (VIF < 2.5). All data collected in summer term (May–June) 2014 at *n* = 6 schools, Essex, UK.

## Appendix A Supplementary methods and questionnaire

Supplementary material. Image 2
