# Supplementary material for: Media device ownership and media use: Associations with sedentary time, physical activity and fitness in English youth
Source: Prev Med Rep. 2016 Jun 3;4:162–8. doi: 10.1016/j.pmedr.2016.05.013 (PMC4929126; doi:10.1016/j.pmedr.2016.05.013)
Supplement: Supplementary file 2 — Supplementary material. [file mmc2.docx]

**Approach and Additional Methodology**

There are many difficulties in researching the associations between use of media technology and the health-related behaviors of young people using traditional research technique and infrastructure. Research is typically designed by adults with little insight into the media habits and trends in youth. Funding applications, research deployment and reporting cycles are measured in (typically 2-3) years while advances in technology are much shorter; hardware updates are often annual, software innovations faster still. vIn an attempt to address the limitations of existing studies in our attempt to investigate the above points, we adopted a novel approach to the design and deployment of this research. We used established protocols and tools to measure outcome measures of physical activity and fitness. To assess the independent variables of interest (sedentary behaviours, media use behavior and media device ownership) we devised our own instruments following consultation with the population of interest. We held initial focus groups (15-17 year olds) to identify suitable menu-choices. These were refined by undergraduate students (18-20 years) before piloting by final year students (20-21 years). This process was completed under the guidance of the lead researcher (40 years) but feedback and comments from piloting populations were used to amend items when agreed by the researchers (in consultation with younger 18-21 years) adults

*Modifications from Pilot Testing*

During pilot testing the initial listing of ‘MySpace’ was removed as being outdated. The reference to ‘Facebook’ as an example of social media was deemed restricting (Facebook was not the most-commonly used social media application in the target population and was referred to as ‘old fashioned’ or ‘for parents’). We removed of the terms ‘video games’ as this term was deemed outdated and referred only to ‘Gaming’. Initial suggestions were that email should be removed (also deemed outdated) form of communication but email was re-instated by the group after discussion with teachers (email communication is used by schools). Both groups also advised of the need for clear differentiation between smart phone and any other mobile phone. This process helped to clarify our definition of device ‘ownership’ – which we differentiated from having access to a device.

Figure S1a (boys) b (girls). Contribution of different behaviours to weekday sedentary time in schoolchildren from six schools in the East of England Data collected in Summer (May-July) term of 2014.

1. Boys

| Using any Computer for Leisure   1. Girls. |  |
| --- | --- |

Using any Computer for Leisure

*Deployment and Validation*

In all, the time from writing to deploying the questionnaire used in this study was 10 weeks. It was not, therefore, possible to fully validate the items used against a known standard. Given the pace of technological innovation and the speed of uptake by young people we do not believe such a standard (apart from direct observation) exists. To determine the credibility of our estimates of total sedentary time we assessed subgroups from two schools using 7-day accelerometry (Table S1). We divided the accelerometer-derived recordings into weekends and weekdays – the latter was further subdivided into ‘in school’ and out of school epochs.

**Table** S1. Sedentary behaviour in sub-sample (n=76, 48% boys) assessed by Accelerometery.

| **Characteristics** | **All (n=76)** | **Boys (n= 37)** | **Girls (n=39 )** |
| --- | --- | --- | --- |
|  | **Mean**  **[SD]** | **mean**  **[SD]** | **mean**  **[SD]** |
| **Sedentary Behaviour**  **Inside School (360 min)** | 295  [20] | 285  [19] | 305  [15] |
| **Sedentary Behaviour**  **After School (90 min)** | 62  [9] | 59  [9] | 64  [8] |
| **Evening Sedentary Behaviour**  **(330 min)** | 258  [18] | 252  [19] | 263  [16] |
| **Weekday Sedentary Time (min)** | 471  [111] | 457  [110] | 484  [113] |
| **Weekends Sedentary Time**  **(min)** | 458  [127] | 436  [117] | 479  [134] |
| **Mean Daily Sedentary Time**  **(min)** | 467  [97] | 451  [91] | 482  [101] |

Sedentary behaviour corresponds to <100 counts per minute.

Data recorded using Actigraph GXT1 uniaxial accelerometer worn for 7-days including data from at least 4 school days as well as Saturday and Sunday. Data collected in summer 2014 in two schools

Correlation between self-reported sedentary time r=0.56, p<0.001 (Weekdays) r=0.41, p=0.001

(Weekends). Correlation of MVPA (not shown) with PAQ-C score for overlapping 7-day epoch r=-0.46, p<0.001.

**Extract of Sedentary Time and Media Ownership and Use Questionnaire**

1. **On a school-day, how much time do you spend sitting down doing the following:**

Time sitting at school (school day is ~6 Hours) ___ Hours ___ min

Time sitting travelling ___ Hours ___ min

Time sitting watching TV or films ___ Hours ___ min

Time sitting doing homework ___ Hours ___ min

Time sitting using any computer for leisure ___ Hours ___ min

Time sitting eating / reading /chatting /doing nothing ___ Hours ___ min

How long do you usually spend sitting down on a Saturday?  ___ Hours ___ min

Time sitting travelling ___ Hours ___ min

Time sitting watching TV or films ___ Hours ___ min

Time sitting doing homework ___ Hours ___ min

Time sitting using any computer for leisure ___ Hours ___ min

Hour sitting eating / reading /chatting /doing nothing ___ Hours ___ min

How long do you spend sitting down on a Sunday? ___ Hours ___ min

Time sitting travelling ___ Hours ___ min

Time sitting watching TV or films ___ Hours ___ min

Time sitting doing homework ___ Hours ___ min

Time sitting using any computer for leisure ___ Hours ___ min

Hour sitting eating / reading /chatting /doing nothing ___ Hours ___ min

1. **Which of the following devices do you have access to at home? (Tick all)**

| TV |  |
| --- | --- |
| DVD or Blu-Ray player |  |
| TVO, Freesat, Skybox or other TV recording device |  |
| Games Console |  |
| Wii or Interactive device i.e Kinect / Play station move |  |
| Desktop computer |  |
| Laptop computer |  |
| Tablet computer |  |
| Smart Phone |  |
| Other mobile phone |  |

1. **Which of the following devices do you *own* personally? (Tick all)**

| TV |  |
| --- | --- |
| DVD or Blu-Ray player |  |
| TVO, Freesat, Skybox or other TV receiving/recording device |  |
| Games Console |  |
| Interactive device i.e Kinect /PlayStation Move |  |
| Desktop computer |  |
| Laptop computer |  |
| Tablet computer |  |
| Smart Phone |  |
| Other mobile phone |  |

*In this case, owning the device means its ‘yours’. You either keep the device in your bedroom or other personal space at home. You may have bought the device yourself or been given it as a present.*

1. How many emails do you send each day (circle ONE answer)

None (0) 5 10 15 20 25 30 35 40 45 50 >50

1. How many instant messages do you normally send each day (circle ONE answer)

None (0) 5 10 15 20 25 30 35 40 45 50 >50

1. How many text messages do you normally send each day (circle ONE answer)

None (0) 5 10 15 20 25 30 35 40 45 50 >50

1. Do you use social media? YES / NO
   1. If **Yes**: How long do you spend using social media on a normal day

<30 min 30-60 min 60-90 min 90 min-2 hours >2 hours
